# Exponential increases in high-temperature extremes in North America

**DOI:** 10.1038/s41598-023-41347-3

**Published:** 2023-11-06

**Authors:** Ali Davariashtiyani, Mohsen Taherkhani, Seyyedfaridoddin Fattahpour, Sean Vitousek

**Affiliations:** 1 Department of Civil, Materials, and Environmental Engineering, University of Illinois Chicago, Chicago, IL USA; 2 Department of Civil and Construction Engineering, Oregon State University, Corvallis, OR USA; 3 Currently employed at Pacific Coastal and Marine Science Center, U.S. Geological Survey, Santa Cruz, CA USA

**Keywords:** Climate change, Environmental impact, Natural hazards

## Abstract

Global warming in the 21st century will alter the frequency of extreme climatic events, such as high-temperature anomalies and “heat waves”. Observations of extreme high temperatures during recent decades have detected upward trends in their frequency of occurrence, and recent state-of-the-art Global Climate Models (GCMs), e.g., Climate Model Intercomparison Projects (CMIPs), notably CMIP5 and CMIP6, have predicted acceleration of temperature trends and high-temperature events by 2100 under projected greenhouse-gas emission scenarios. Hence, the 21st century is expected to experience substantial shifts in the occurrence of extreme events, where present-day, extreme-but-rare high-temperature events will become common during the summer months. The increasing frequency of extreme heat may affect the health and resiliency of social, biological, and infrastructure systems in many regions worldwide, underscoring the need for accurate and reliable long-term assessments of climatic change across global and regional scales. So far, many investigations of high-temperature extremes have been carried out under end-point scenarios, e.g., by comparing GCM-projected changes in the frequency of high-temperature extremes expected in the late 21st century to the late 20th century. In this study, we use extreme value theory and decades of observations of high-temperature extremes at thousands of meteorological stations across North America to investigate continuous shifts in the frequency of extreme high-temperature events due to projected local warming trends. We find that the odds of exceedance of 50-year extreme high-temperature events increases exponentially with increases in mean local temperature. At a majority of the stations studied here, a local mean temperature increase of 0.5–1 $^{\circ }$C can double the odds of exceedance of 50-year extreme high-temperature events. Based on time-dependent temperature projections, the odds of exceedance of 50-year extreme high-temperature events doubles approximately every 20 years (or sooner) for $\sim$ 96% of the stations. Moreover, we find that, for $\sim$ 80% of the stations in North America, investigated here, the 50-year extreme high-temperature events will be exceeded annually before 2100.

## Introduction

Earth’s global average surface temperature has risen 1.09 $^{\circ }$C (1.59 $^{\circ }$C over land regions) since pre-industrial levels^1^. This rise is linked to the growing concentration of atmospheric greenhouse gases^1,2^, predominantly from anthropogenic sources since the mid-20th century^3–7^. Increased concentrations of greenhouse gases and global warming trends also affect extreme climatic events^8,9^, such as high-temperature extremes, which can have a multitude of ecological and societal impacts^3,10–13^.

A simple increase in mean temperatures can lead to increases in the frequency and intensity of extreme temperature events^10,13,14^. Further, anthropogenic emissions^1,2,15,16^ can also drive fundamental, non-linear changes to extreme climatic events (i.e., changes in the skewness and tail behavior of probability distributions for extreme temperatures, for example). In a warming climate, the occurrence of extreme high-temperature events is expected to increase with time, such that rare (e.g., decadal and longer return period) events can become common (e.g., annual) in the future^17^.

Investigations of extreme temperature events in the literature are primarily assessed via two different approaches: (1) climate-extreme indices, which represent pre-defined, relatively frequent annual or monthly climatic events, and (2) extreme-value theory (e.g., refs.^18^), a branch of statistics to model extreme events and their probability distributions, typically using historical observations^3,10,11,19–24^.

Global Climate Models (GCMs) have played a central role in assessing historical and future changes in global and regional climatic extremes, including temperature extremes^21^. Among them, the popular Coupled Model Intercomparison Project Phase 5 (CMIP5^25^) and Phase 6 (CMIP6^26^), coordinated by the World Climate Research Programme (WCRP), have projected increases in the frequency and intensity of high-temperature extremes and extended heat waves under the majority of emission scenarios (e.g., refs.^1,22,27–32^). For the contiguous U.S., a significant increase in the frequency of extreme high temperatures has been observed during recent decades^33,34^, and a persistent upward trend in the frequency of high-temperature extremes and heat waves is projected by the end of the 21st century^24,35–38,^.

Many studies investigating the transformation of extreme high-temperature events (e.g., refs.^22,27^) have focused on end-point scenarios, e.g., comparing GCM-derived late 21st-century extreme high-temperature events to late 20th-century events. Fewer studies have investigated the continuous trend in the frequency of extreme high-temperature events in the coming decades. Here, we use stationary Generalized Extreme Value (GEV)^18^ and empirical probability distribution based on decades of historical temperature observations to demonstrate an exponential increase in the frequency of extreme high-temperature events throughout Northern America (U.S., Canada, and Mexico) driven by local temperature projections under a range of emission scenarios (i.e., the ensemble low-end, intermediate, and high-end Representative Concentration Pathway [RCP] 8.5^4,39^).

The remainder of the manuscript is divided into four sections. We present the results of this study in section “Results”, discuss them in section “Discussion”, and conclude in section “Conclusions”. Outside of the main body of the article, section “Methods” elaborates on the methodology and temperature data used in our analysis.

## Results

**Figure 1:**
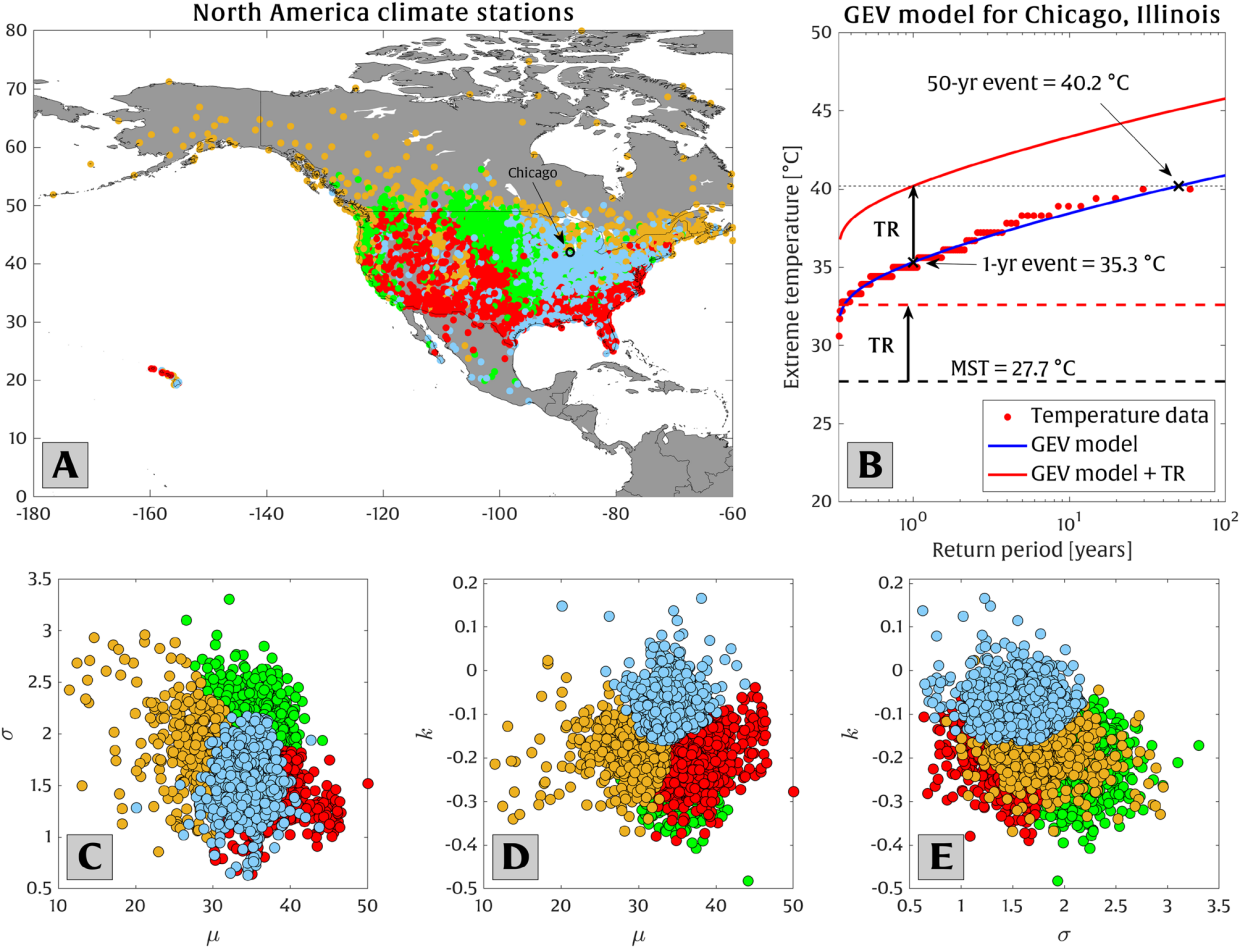
(**A**) The network of climate stations used in the current study and (**B**) an example of the recorded extreme high-temperature data (red dots) and the GEV fit (blue line) for a single climate station in Chicago, Illinois, subject to a hypothetical future temperature rise (TR). Mean summer temperature (MST) is shown in the black dashed line in panel (**B**). Panels (**C**)–(**E**) present the “pair plots” displaying the relationship between the three best-fit parameters of each GEV distribution ($\mu$, $\sigma$, and *k*) for the 4266 stations investigated here. The stations are clustered into four groups (blue, green, orange, and red) representing different combinations of their GEV distribution parameters.

Our study analyzes 4266 long-standing climate stations in North America (shown in Fig. 1A), of which 3,737 are located within the U.S., while 497 and 32 stations are in Canada and Mexico, respectively, using data and techniques described in section “Methods”. North America is chosen as the focus region for the current study due to its rich availability of high-quality temperature data (e.g., the density of climate stations and the high frequency/long duration of recorded temperature data). Additionally, North America possesses a diverse but manageable number of micro-climates for meaningful clustering analysis (see Fig. 1C–E). Finally, North America generally exhibits higher mean temperature projections by 2100 [$\sim$ 3.9–7.3 $^{\circ }$C] than the global average [$\sim$ 2.8–5.2 $^{\circ }$C] under a range of RCP 8.5 emission scenario projections (where 2000 is the baseline year; see Extended Data Fig. 1).

Figure 1B shows an example of a Generalized Extreme Value (GEV) distribution fitted to observations of the top three annual maxima of daily maximum temperature (at Chicago, Illinois, U.S.). Figure 1C–E show four clusters (obtained via K-means clustering method^40^) of the GEV parameter estimates (i.e., $\mu$, $\sigma$, and *k*, which represent proxies for the mean, standard deviation, and tail behavior, respectively). Although the stations are not directly classified/clustered based on their geographic locations, the clustering analysis reveals consistent geographical patterns in the GEV parameters, which are driven by the underlying patterns of temperature extreme across North America (e.g., stations colored in red generally occur at low- to mid-latitudes and exhibit extreme high temperatures with low inter-annual variability, as discussed in more detail in section “Discussion”).

**Figure 2:**
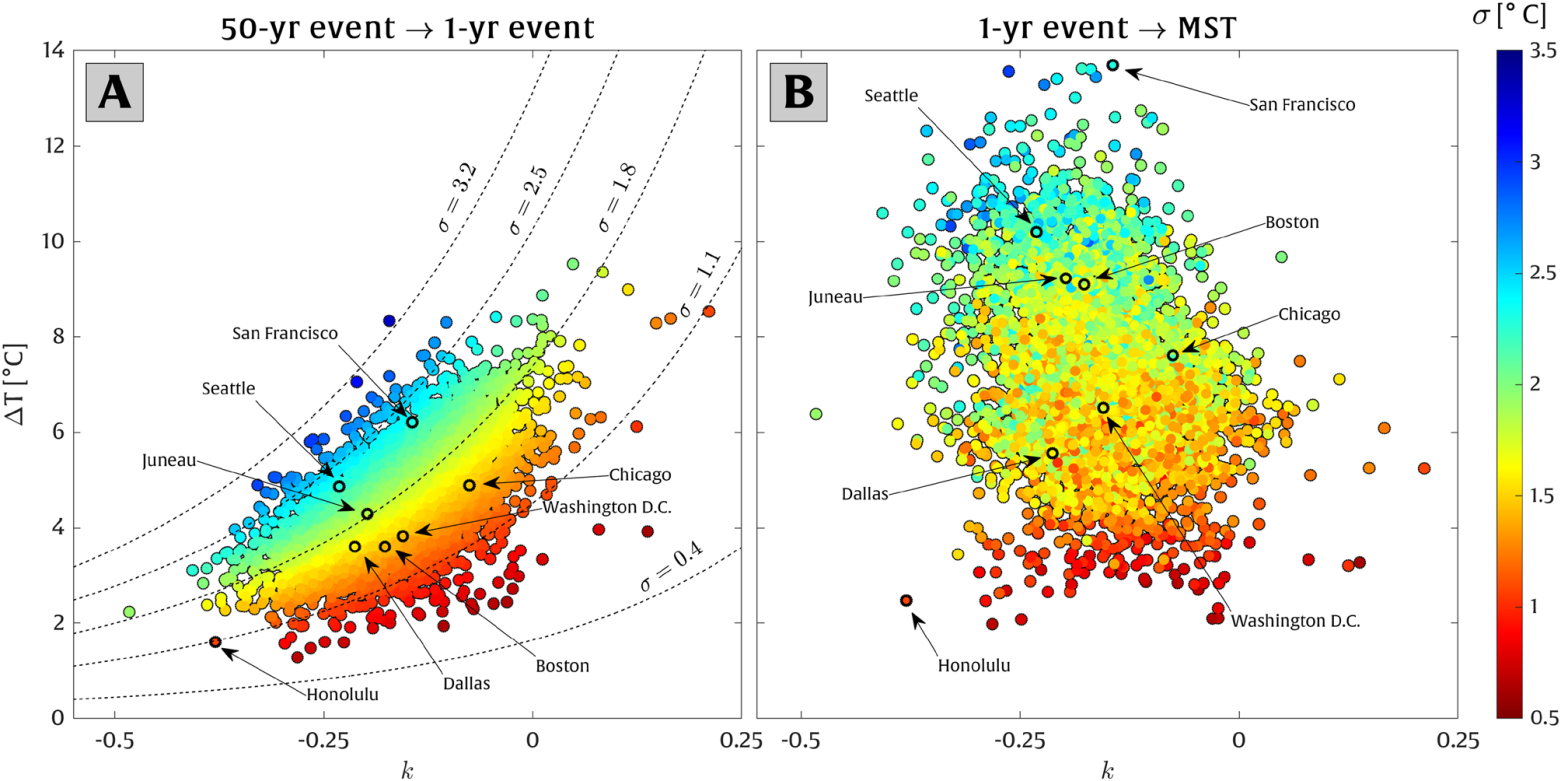
Differences in extreme temperatures (i.e., $\Delta T$ metrics) for 4,266 climate stations across North America (where some stations in major cities are labeled). (**A**) The $\Delta T_{50yr\rightarrow 1yr}$ metric represents the temperature difference between the 50-year and the 1-year extreme high-temperature event, and (**B**) the $\Delta T_{1yr\rightarrow MST}$ metric represents the temperature difference between the 1-year event and the mean summer temperature. The metrics are displayed along with their corresponding GEV shape parameter (*k*) on the x-axis and the scale parameter ($\sigma$), which is color-coded according to the color bar.

Using the GEV distributions, whose parameters are shown in Fig. 1, we calculate the differences between the 50-year and 1-year extreme temperatures, denoted by $\Delta T_{50yr\rightarrow 1yr}$ (as shown in Fig. 2A, which is calculated using Eq. (12)). We target $\Delta T_{50yr\rightarrow 1yr}$ as a metric to characterize severe global warming impacts since it expresses the transformation from a “once-in-a-lifetime” event to an annual event. Furthermore, we consider the difference between the 1-year (annual) extreme temperature event and the mean summer temperature (MST), denoted by $\Delta T_{1yr\rightarrow MST}$ (as shown in Fig. 2B, which is calculated using Eq. (13)). This metric is significant as it projects a future where the “once-per-summer” extreme temperatures could become the norm during summers, creating severe and persistent high-temperature conditions that pose serious challenges to human habitation in certain locations.

The $\Delta T$ metrics, shown in Fig. 2, can be compared to projections of local warming trends. For example, a projected temperature rise equivalent to $\Delta T_{50yr\rightarrow 1yr}$ would cause the present-day 50-year temperature event to occur every year (in an otherwise stationary climate system). For instance, Fig. 1B shows the difference between the $T_R$ = 50-year temperature event (40.2 $^{\circ }$C) and the $T_R$ = 1-year temperature event (35.3 $^{\circ }$C) at Chicago, which is equivalent to $\Delta T_{50yr\rightarrow 1yr}=4.9$ $^{\circ }$C. Hence, a projected temperature increase of about 4.9 $^{\circ }$C (which is anticipated by 2100 according to the intermediate RCP 8.5 scenario for Chicago; see Extended Data Fig. 1) could cause present-day “once-in-a-lifetime” temperatures to be exceeded annually. Fortunately, our projections suggest that for a majority of stations, the condition represented by $\Delta T_{1yr\rightarrow MST}$ is not likely to occur in the next few decades, except at a limited number of stations. However, the consideration of this scenario is important to understand the potential long-term impacts of climate change.

Figure 2 also depicts how the $\Delta T_{50yr\rightarrow 1yr}$ and $\Delta T_{1yr\rightarrow MST}$ metrics are affected by the GEV shape parameter, *k*, and scale parameters, $\sigma$, across each observation station. Each station is color-coded by its corresponding scale parameter, $\sigma$, which is often associated with the station’s inter-annual variability and its vulnerability to rapid increases in the frequency of extreme events due to local warming trends (as discussed in more detail in section “Discussion”). Parameter *k* has a positive correlation with the $\Delta T$ metrics, i.e., increasing *k* increases $\Delta T$ (Fig. 2), but only a small minority of the stations (1.4%) have a positive shape parameter ($k>0$). Parameter $\sigma$ is also positively correlated with the $\Delta T$ metrics, i.e., increasing $\sigma$ increases $\Delta T$ (Fig. 2). By comparing the two metrics in Fig. 2, we observe a more obvious relationship with the GEV parameters in $\Delta T_{50yr\rightarrow 1yr}$ than in $\Delta T_{1yr\rightarrow MST}$. However, this is perhaps not surprising considering the fact that the GEV parameters uniquely describe the $\Delta T_{50yr\rightarrow 1yr}$ metric, but the $\Delta T_{1yr\rightarrow MST}$ metric is affected by the site’s mean summer temperature, which can be somewhat unrelated to the GEV parameters, given that the GEV distribution only parameterizes the occurrence of extreme temperature events, which are generally well above mean summer temperatures.

We find that the difference between the 50-year event and the 1-year event is less than 5 $^{\circ }$C for 70% of the climate stations in Fig. 2. This portion increases to more than 95% of stations for which the differences between the 50-year event and the 1-year event are less than 6.5 $^{\circ }$C. The average temperature difference between the 1-year event and MST is 7.4 $^{\circ }$C (with a standard deviation of 1.9 $^{\circ }$C). For the sake of comparison, note that the RCP 8.5 scenario projects an average temperature rise of $\sim$ 5.7 $^{\circ }$C ($\sim$ 4 $^{\circ }$C) in North America (globally) by 2100.

**Figure 3:**
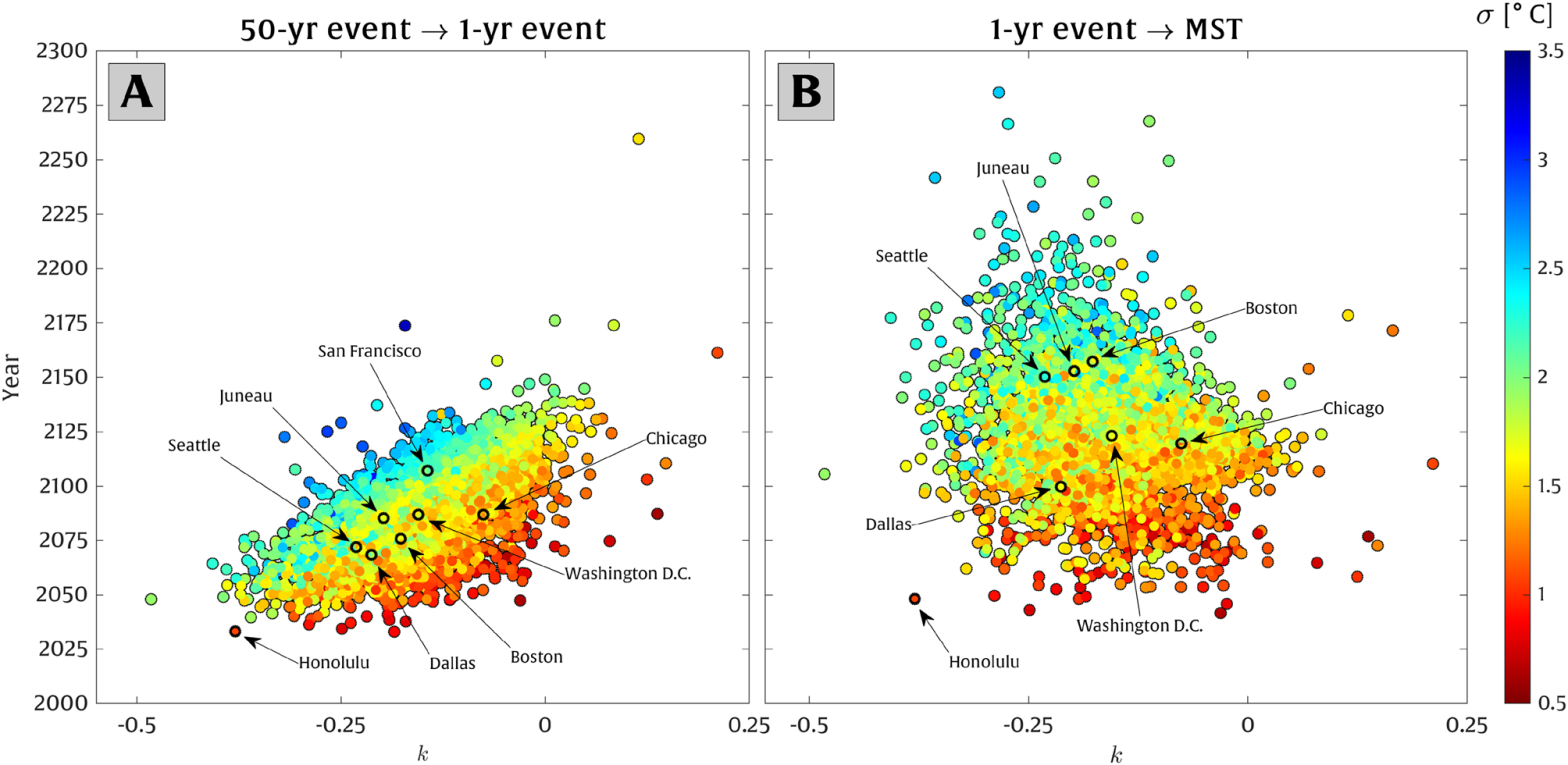
The anticipated year for regime shifts in return periods of extreme temperature events under the intermediate RCP 8.5 emission scenario. Panel (**A**) represents the year when the present-day 50-year extreme temperature occurs annually (in an otherwise stationary climate). Similarly, panel (**B**) shows the year when the present-day annual extreme events become the mean summer temperature. A few notable climate stations in major cities are labeled. As in Fig. 2, the x-axis represents the GEV shape parameter (*k*), and the color of each station depicts the GEV scale parameter ($\sigma$).

In the following analysis, we apply intermediate RCP 8.5 projections of local temperature rise (described in section “Extreme value theory”) at each climate station to find the year in the future when projections of local temperature increase surpass $\Delta T_{50yr\rightarrow 1yr}$ and $\Delta T_{1yr\rightarrow MST}$ (Fig. 3). As above, time horizons shown in Fig. 3 occur sooner for lower values of $\sigma$ and *k*.

According to the intermediate RCP 8.5 temperature rise scenario, the present-day 50-year extreme temperature events transition to an annual occurrence for more than 80% of the stations by 2100. Washington D.C., Boston, Dallas, Seattle, Chicago, and Honolulu are among the cities projected to undergo such a transformation. As shown in Fig. 3, only $\sim$13% of sites are projected to experience the transition from present-day 1-year events to the present-day mean summer temperature by 2100.

**Figure 4:**
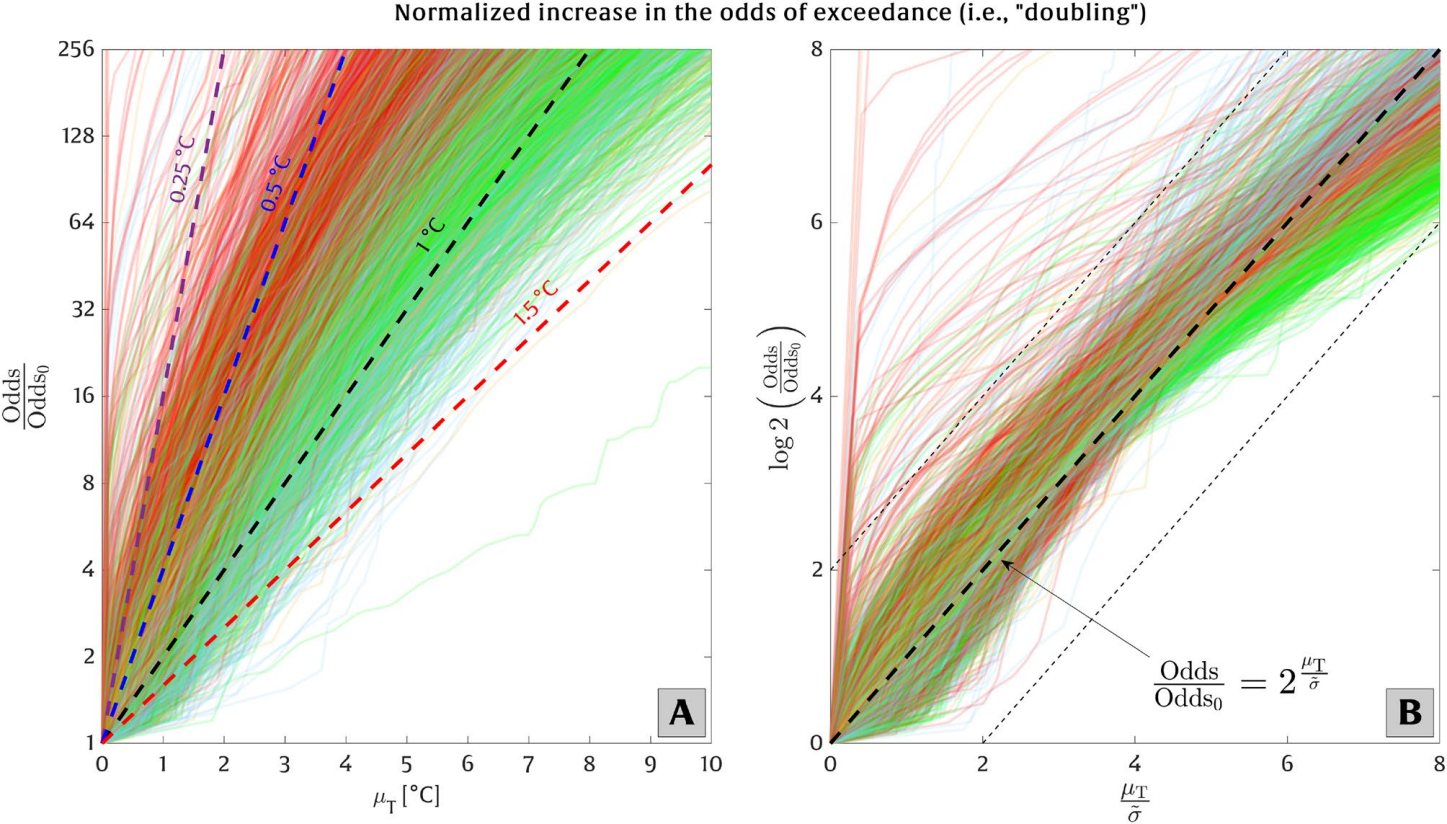
The relationship between local temperature rise ($\mu _{T}$) and the relative increase in the odds of exceeding the present-day 50-year temperature event, $O/O_0$. Each colored solid line corresponds to a single climate station and is colored according to its classification as depicted in Fig. 1 (some transparency is employed to reduce visual blockage of the different lines). Panel (**A**) shows the relative odds ($O/O_0$; on a logarithmic scale) versus temperature rise ($\mu _{T}$; on a linear scale). Accordingly, relationships following a straight line match an exponential growth (i.e., doubling) rate with a fixed amount of temperature rise. The purple, blue, black, and red dashed lines on panel A correspond to the doubling of the odds of exceedance for every 0.25, 0.5, 1, and 1.5 $^{\circ }$C of temperature rise, respectively. Panel (**B**) plots the base-2 logarithm of the relative odds, i.e., $\log _2(O/O_0)$, versus the normalized temperature shift ($\mu _{T}/\widetilde{\sigma }$), where $\widetilde{\sigma }$ is determined empirically from the slope of each curve in panel (**A**). The bold dashed line on panel (**B**) represents Eq. (8), and the thin dashed lines enclose regions within a factor of four of the relationship in Eq. (8).

**Figure 5:**
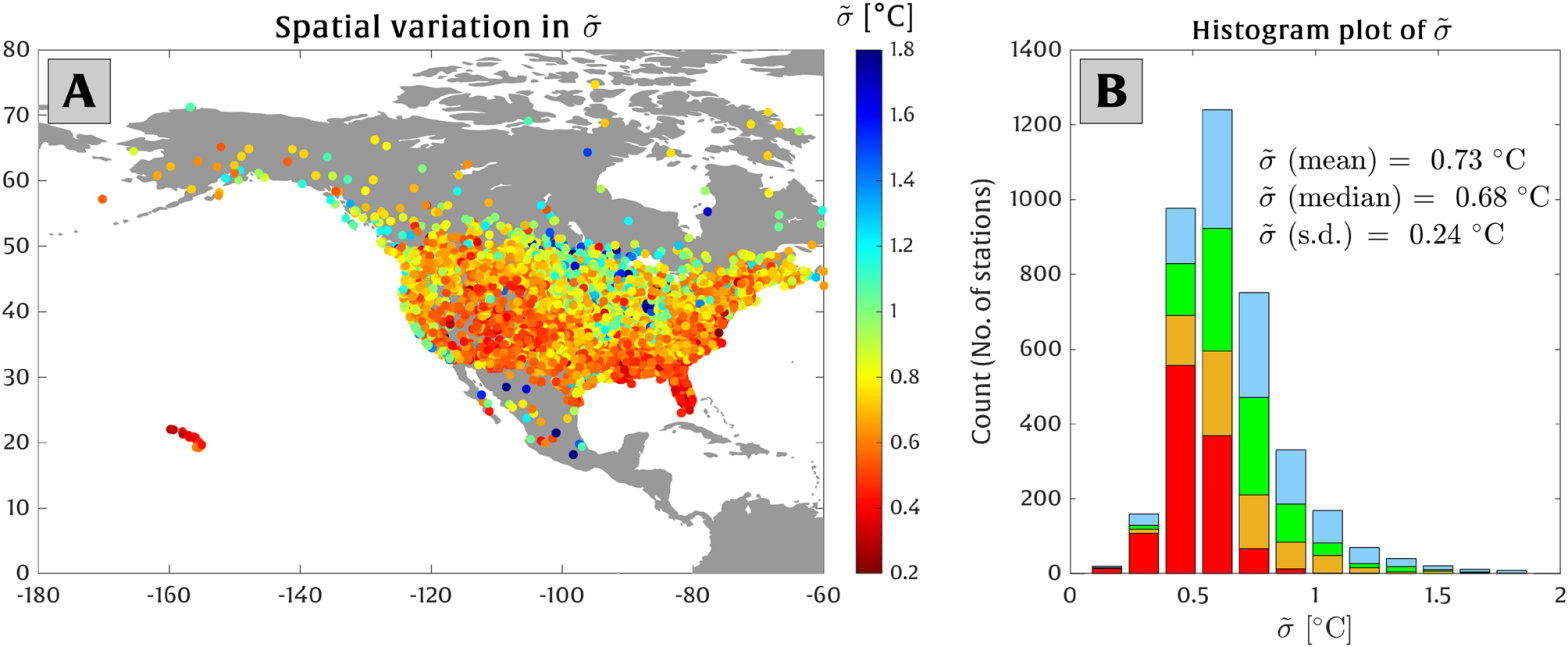
Variability of the local temperature scale ($\widetilde{\sigma }$) that doubles the odds of exceeding the present-day 50-year extreme temperature event. Panel (**A**) depicts the geographical distribution of $\widetilde{\sigma }$ for all observation stations analyzed here, and panel (**B**) displays the histogram plot of $\widetilde{\sigma }$. The colors on the histogram bars in panel (**B**) represent the clusters shown in Fig. 1.

So far, our results provide some insight into extreme temperature characteristics and the time horizon for regime transitions among extreme thresholds, but they do not explain how the effects of temperature rise manifest continuously over time. In Figs. 4 and 5, we investigate the continuous growth in the frequency of extreme temperatures caused by a shift in mean temperature (i.e., $\mu _T$). To this end, we analyze “odds of exceedance” (*O*), which is a close alternative to the “probability of exceedance” (*E*) and is calculated via Eq. (6) [$O=E/(1-E)$]. We evaluate increases in the odds of exceedance of extreme temperature events ($O/O_0$) using the simplified expression in Eq. (8) [$O/O_0=2^{{\mu _T}/\widetilde{\sigma }}$, where $\widetilde{\sigma }$ is a parameter that is equivalent to a required local shift in mean temperature to double the odds of exceedance]. To calculate the parameter $\widetilde{\sigma }$, we apply a shift in the empirical odds distribution, according to Eq. (7) in section “Methods”, and fit a trend line to the resulting curve (see Fig. 4A).

To model continuous rates of growth (which are idealized by the doubling parameter $\widetilde{\sigma }$), we favor the use of the odds of exceedance variable over the probability of exceedance variable. As discussed in Taherkhani et al.^41^, the odds of exceedance can take on any value from zero to infinity, thus making it particularly well-suited to model growth rates, whereas the exceedance probability is less suitable due to its upper bound of $E=1$. The advantage of applying the odds of exceedance is evident for higher temperature shifts (i.e., higher $\mu _T$ values) when comparing Extended Data Figs. 2 and 3, and also Extended Data Figs. 4 and 5, where Extended Data Figs. 2 and 4 use the probability of exceedance (i.e., $E/E_0$) and Extended Data Figs. 3 and 5 employ the odds of exceedance (i.e., $O/O_0$) in representing the above-mentioned continuous growth.

Figure 4 shows the relationship between the temperature rise and the relative increase in the odds of exceeding the current 50-year extreme temperature event, $O/O_0$. The x- and y-axes of Fig. 4A are plotted on linear and logarithmic scales, respectively, meaning that relationships that resemble straight lines exhibit an exponential growth rate in the odds of occurrence with respect to local temperature rise. Each solid line in Fig. 4A corresponds to a single climate station investigated here, which is color-coded based on its classification shown in Fig. 1. The purple, blue, black, and red dashed lines are computed according to Eq. (8) and represent a doubling of the odds of exceedance of the 50-year extreme temperature event under every 0.25, 0.5, 1, and 1.5 $^{\circ }$C of temperature rise, respectively. This means that, for locations that are particularly susceptible to extreme temperature events (such as the red-colored stations in Fig. 4A), a slight rise in mean temperature can lead to a substantial increase in the odds of exceedance. Fig. 4B depicts the base-two logarithm of the relative odds of exceedance as a function of the normalized local temperature rise ($\mu _{T}/\widetilde{\sigma }$), recalling that $\mu _{T}$ is the shift in local mean temperature and $\widetilde{\sigma }$ is the doubling parameter, which is derived empirically from the average slope of the curves in panel A. Note that the amount of temperature rise required for the doubling of the odds of exceedance ($\widetilde{\sigma }$) at any station is not the same as the GEV scale parameter ($\sigma$), although these values are closely related under certain conditions (e.g., when $k\approx 0$; see Extended Data Fig. 6). The analysis shown in Fig. 4B is an attempt to normalize the rate of growth in the odds of exceedance using Eq. (8). The thick black dashed line on panel B plots the relationship based on Eq. (8), and curves closer to this black line will more closely follow the simplified form of exponential growth of their odds of exceedance. We find that approximately 98% of the curves (stations) fall within a factor of 4 of the rate predicted by Eq. (8) (areas enclosed by the thin black dashed lines in Fig. 4B) up to 8 doubling periods, which is equivalent to a $2^{8}$-fold (256-fold) increase in the odds of exceedance.

In this study, our findings are based on the empirical probability distribution of temperature events, i.e., $E_0(x)$ and $O_0(x)$. Since the empirical distributions are non-parametric, the growth rates are not controlled by the behavior of any specific statistical model, such as GEV. Additionally, we utilize empirical distributions of all recorded temperatures instead of a distribution that only considers extreme temperatures, which assists in examining the transition from infrequent to common events. We show (in Extended Data Figs. 2 and 3) that the exponential growth rates are relatively insensitive to the form of the exceedance distribution when applying three different forms of probability distributions of extreme temperature events, i.e., (1) the empirical probability distribution of all recorded temperatures, (2) the empirical distribution of extreme temperature observations, and (3) the GEV model fitted to observations of extreme temperatures.

Figure 5 depicts the spatial variability across North America of $\widetilde{\sigma }$, i.e., the local temperature rise that doubles the odds of experiencing the present-day 50-year extreme temperature threshold. At most of the locations, $\widetilde{\sigma }$ varies in the range of $\sim$ 0.4–1.3 $^{\circ }$C (2.5 and 97.5 percentiles) with an average of 0.73 $^{\circ }$C and a standard deviation of 0.24$^{\circ }$C (Fig. 5B).

**Figure 6:**
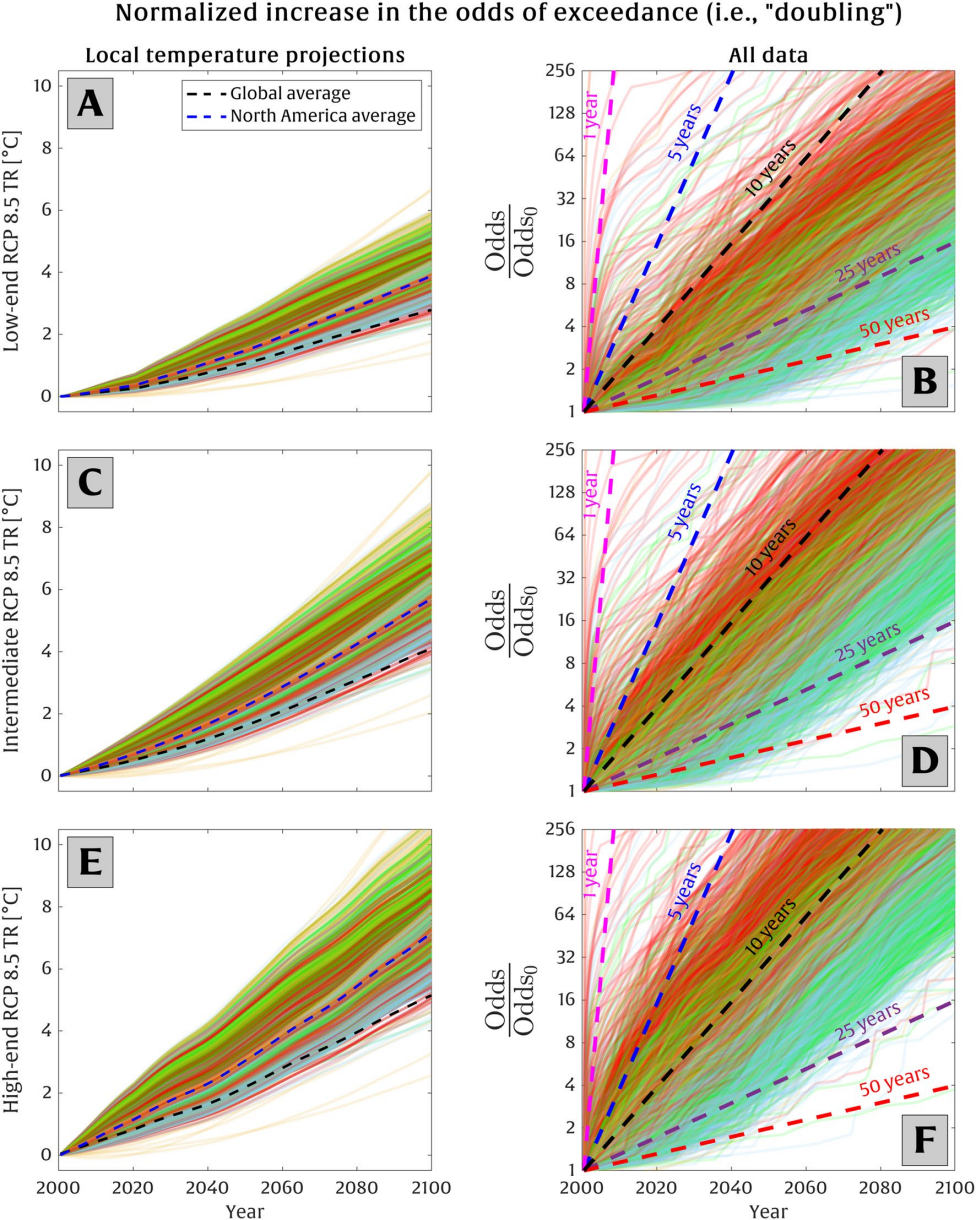
The increase in the odds of exceeding the present-day 50-year threshold versus time. Here, the local temperature-rise projections are provided by KNMI^42^ and WDCC^43^, and low-end, intermediate, and high-end RCP 8.5 scenarios are shown in panels (**A**), (**C**), and (**E**), respectively. The colored lines in panels (**A**), (**C**), and (**E**) demonstrate the local temperature rise projections under each RCP 8.5 scenario applied to each station, while the black and blue dashed lines in these panels represent the global and North America average projections, respectively. Panels (**B**), (**D**), and (**F**) illustrate the relationship between the relative odds of exceedance of a 50-year extreme temperature event versus time at a given station. The use of a logarithmic scale on the y-axis indicates that linear relationships on panels (**B**), (**D**), and (**F**) correspond to exponential growth (i.e., doubling) over a fixed period of time on the x-axis. The pink, blue, black, purple, and red dashed lines denote a doubling rate of the odds of exceedance every 1, 5, 10, 25, and 50 years, respectively, according to Eq. (9). Each colored line in these panels represents an individual station and is color-coded based on its classification shown in Fig. 1 (with some transparency applied for better visualization).

Figure 6 (panels B, D, and F) presents the continuous increase in the odds of exceedance of the present-day 50-year extreme temperature event ($O/O_0$) at each station as a function of time under a range of RCP 8.5 scenarios. Panels A, C, and E in Fig. 6 correspond to the low-end, intermediate, and high-end RCP 8.5 scenarios, respectively, for the local projections of temperature rise at all stations considered. Each curve corresponds to the results for a single station, which is colored according to its associated cluster shown in Fig. 1. The dashed black and blue curves in panels A, C, and E represent the average of the global and North America temperature projections, highlighting that North America’s warming trend outpaces the global trend. In panels B, D, and F, the pink, blue, black, purple, and red dashed lines correspond to a doubling in the odds of exceedance every 1, 5, 10, 25, and 50 years into the future, respectively. As in Extended Data Figs. 2 and 3, we compare three different forms of probability distributions of extreme temperature events, $E_0(x)$ and $O_0(x)$, and find that the growth rates are largely insensitive to the form of the distribution used (see Extended Data Figs. 4 and 5, respectively). In general, we find that North America exhibits more rapid, exponential growth in the frequency of extreme temperature events with increasing temperature-rise projections (i.e., for high-end RCP 8.5 projections compared to low-end projections).

**Figure 7:**
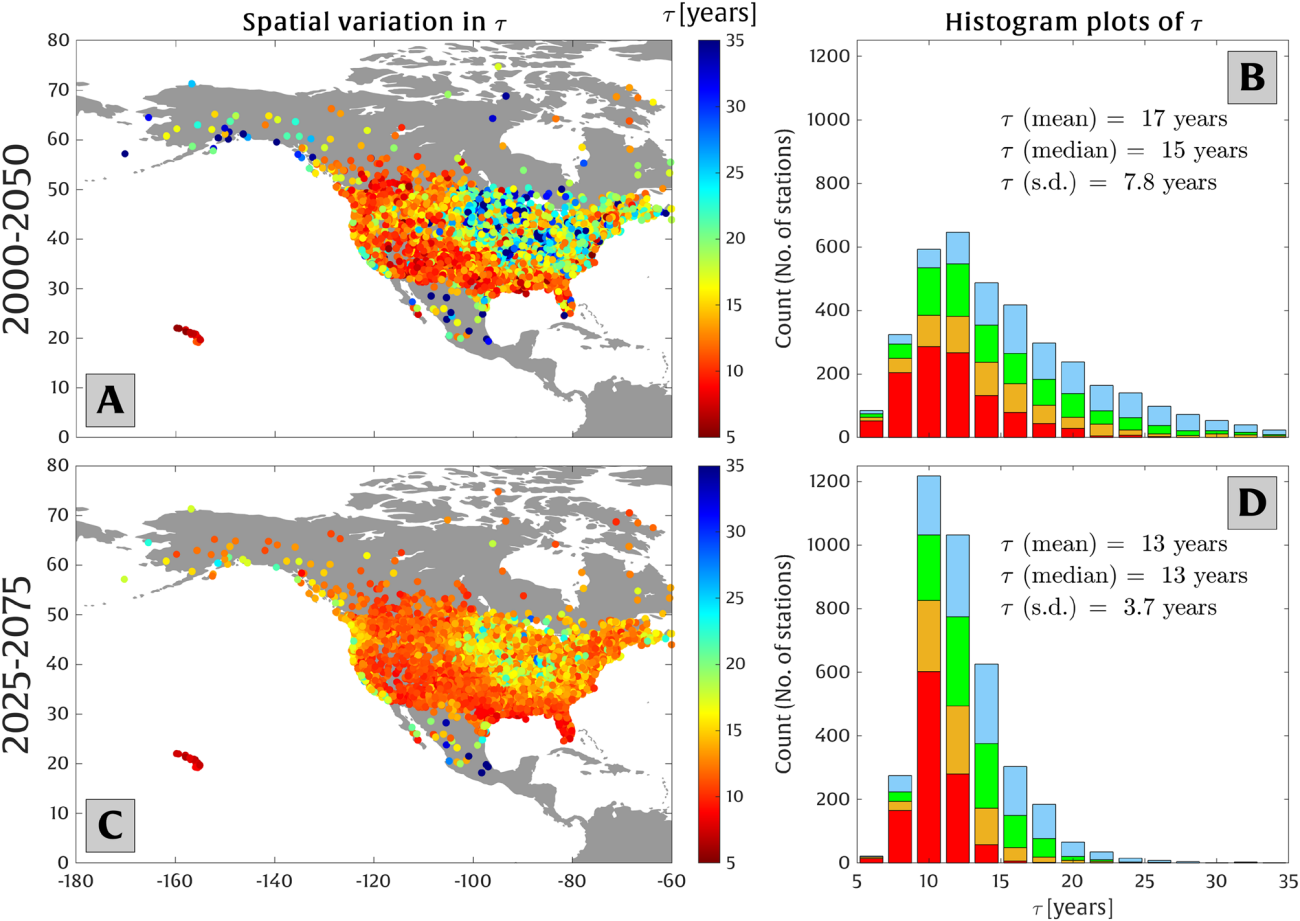
The spatial and temporal variability of the doubling time scale ($\tau$) over which local temperature rise doubles the odds of exceedance of a present-day 50-year extreme temperature event (see Eq. (9)). Panels (**A**) and (**C**) show the spatial variability of ($\tau$) at all climate stations across North America under the intermediate RCP 8.5 scenario for the time periods 2000–2050 and 2025–2075, respectively. Panels (**B**) and (**D**) show histogram plots of $\tau$ for these time periods. Similar to Fig. 5, the colors on the histogram bars in panels (**B**) and (**D**) represent the clusters shown in Fig. 1.

Finally, we analyze the spatial variability of the doubling time scale, $\tau$, which quantifies the amount of time required for projections of local temperature to double the odds of exceeding the present-day 50-year extreme temperature event (based on Eq. 9). This metric is calculated empirically by fitting trend lines to the curves shown in Fig. 6B. We examine the temporal variability of $\tau$ across all observation stations in North America during two different time periods: 2000–2050 and 2025–2075. These time periods were chosen to represent the current/near-term state of climate and the future state where temperature rise accelerates, respectively. The results, presented in Fig. 7, show that the mean value of $\tau$ is $\sim$17 years for the 2000–2050 period and decreases to $\sim$13 years for the 2025–2075 period due to the accelerated global and local warming. The histogram colors in panels B and D in Fig. 7 correspond to the stations’ cluster (shown in Fig. 1) based on their GEV model parameters.

## Discussion

Regional and local changes in air surface temperature can differ considerably from global trends^44^. For instance, due to regional variability^22^, a 2 $^{\circ }$C globally averaged temperature increase can correspond to at least 3 $^{\circ }$C of local warming for much of the Northern Hemisphere^45^. Thus, it is advantageous to investigate sub-regional changes in mean and extreme temperatures, as in the current study, in order to develop community-specific response plans^45–47^.

Many studies investigating changes in temperature extremes do not assess the continuous shift in the frequency of temperature extremes. For example, Kharin et al. (2013)^27^ identified the increase in the frequency of 20-year temperature extremes by comparing historical (1986–2005) and projected (2081–2100) extremes via CMIP5 models, and similarly, Li et al. (2021)^22^ found changes in the frequency of 50-year temperature extremes, by comparing periods of 1985–2014 and 2071-2100 using CMIP6 models. We have taken a slightly different approach to study extreme temperature end-members in the current analysis. As shown in Fig. 2, we investigate the differences in temperature end-members of rare (i.e., 50-year return period) events, annual events, and mean summer conditions using a vast number of historical observations. For most locations, the temperature differences between these end members are comparable in magnitude to projected changes in mean temperature over the 21st century.

Further, in this study, we also examine continuous shifts in temperature extremes by adopting a stationary statistical approach, which assumes that the underlying probability distributions (i.e., in this case, the empirical probability distributions or GEV-modeled probability distributions and their parameters) describing extreme high-temperature events will remain roughly constant over time, except for a shift in the mean caused by local warming. Hence, we do not consider the potential effects of non-stationary, future changes to distribution’s variability (i.e., $\sigma$) or skewness (i.e., *k*), and as such, the current approach is subject to limitations, discussed below.

Stationary statistical models potentially oversimplify the behavior of future extreme climatic events^48^. The underlying assumption of stationarity (e.g., applying a fixed probability over time) may not be entirely valid since climate change can alter the variability and upper bounds (e.g., $\sigma$ and *k* in our GEV analysis, respectively) of extreme events^11,13^. The alternative, non-stationary approach^11,49–51^ allows for the probability distributions (and their associated parameters) to vary with time. However, the non-stationary methods generally require either longer-term historical observations or the use of climate models (i.e., GCMs) to project future extremes. When applying historical observations, the performance of stationary versus non-stationary approaches is problem-specific^52^, although the two approaches often return similar results. Studies that fit model-predicted extreme temperature data, as opposed to historical observations, to obtain extreme value distributions are also subject to sources of error (^26,53^). For example, the model-predicted extreme temperatures may not resolve micro-climates (such as urban heat islands) in ways that they can be resolved using observations. Furthermore, many GCMs do not run continuous simulations but are instead often separated into 10-, 20-, or 30-year-long historical and future projected periods. Also, modes of climate variability (e.g., El Niño/Southern Oscillation (ENSO)) that are historically found to amplify the extreme high-temperature events across North America^54,55^, are a form of non-stationarity that alter the tail behavior of extreme temperature distributions. Capturing the influence of ENSO on temperature extremes would require realistic projections of future ENSO events into the 21st century, which is currently beyond the state of the art of most (if not all) GCMs (see, e.g., refs.^56–58^), rendering the assessment of how future ENSO variability affects the statistics of future extreme temperatures beyond the scope of this study. There are, however, many aspects of extreme high-temperature change that can only be investigated using models. Some evidence indicates that the processes governing the extreme temperatures are partly independent of the ones affecting the mean temperatures; thus, temperature extremes and mean temperatures need not adhere to identical trends^30,59^. Moreover, the relationship between shifts in the mean temperatures and the corresponding changes in the probability of extreme temperatures is inherently nonlinear^14^. Along these lines, it is predicted that changes in extreme high temperatures may outpace the changes in annual mean temperatures over the majority of the land areas globally^22,60^. Nevertheless, the data-driven, stationary statistical approach adopted here can complement the plethora of complex climate model-driven studies^10^, particularly for investigating near-term, continuous shifts in extreme high temperatures over the next several decades, over which time scale the projections of increased regional mean temperatures are more robust/certain compared to the projections of climate modes, such as ENSO.

The vast number of stations analyzed here allows for sub-regional investigations of the behavior of extreme high temperatures. In Fig. 1, we investigate the spatial variability of GEV distribution parameters (i.e., $\mu$, $\sigma$, and *k*) and find significant spatial coherence. As expected, there is a latitudinal dependence on the mean temperature (i.e., $\mu$-parameter). For example, stations with red clusters generally appear at lower latitudes, and stations with orange clusters appear at higher latitudes. Likewise, there is a distinct correlation between latitude and the scale parameter ($\sigma$). Generally, low-latitude regions have smaller values of $\sigma$ (as seen in the red cluster in Fig. 1A). Mid- and high-latitudes cover a broad range of $\sigma$ (as seen in the orange, green, and blue clusters in Fig. 1A). It is well established that lower values of $\sigma$ increase the vulnerability of that location to more frequent extreme events associated with a shift in the mean^61–63^, as in the current study. Interesting patterns in the GEV shape parameter (*k*) are evident across the U.S. Midwest and the Appalachian Mountains regions, as shown in the blue cluster in Fig. 1A, where the *k* values are generally the highest. This is potentially driven by the tendency for anomalously large, yet rare, high-temperature extremes occurring in the blue-clustered region (that is sometimes referred to as the “extreme heat belt”^64^, which hosted the 1995 heat wave in Chicago, Illinois^65,66^, for example).

A very small percentage (1.4%) of our locations, which mostly fall inside of the blue cluster, exhibit positive values of the GEV shape parameter ($k>0$), indicating that their exceedance probability distribution is not bounded. A non-zero probability for arbitrarily high temperatures may not make sense physically but potentially stems from outliers present in the relatively short data sets used in this study. Across much of our analysis (e.g., in Figs. 2 and 3), we find that cities on the East Coast (with smaller $\sigma$ values and mid-range *k* values) represent higher susceptibility to temperature rise impacts compared to cities on the West Coast, which is also evidenced by the higher number of extreme heat waves causing heat-related mortality on the Eastern and Midwestern U.S. historically (e.g., ref.^67^).

The correlation between the cluster of the stations depicted in Fig. 1 and their susceptibility to temperature rise in shifting the extreme temperatures is shown in Fig. 4, where red-cluster stations (generally low-latitude stations) roughly exhibit more vulnerability to warming (i.e., approximately every 0.5 $^{\circ }$C of local temperature rise doubles the odds of exceedance). On the other hand, green, blue, and orange cluster stations (generally occurring at mid/high latitudes) are less vulnerable. There is significant spatial variability in the amount of temperature rise required to double the odds of exceedance throughout the stations investigated here (see Fig. 5). For example, Western and Southeastern U.S. and Hawaii have $\widetilde{\sigma }$ values in the range of $\sim$ 0.2–0.8 $^{\circ }$C. Stations in southern Canada, however, show limited vulnerability with $\widetilde{\sigma }$ values of $\sim$ 1–2 $^{\circ }$C. On average (for all stations), the doubling temperature scale $\widetilde{\sigma }$ is 0.73 $^{\circ }$C across North America.

For the majority of the stations, the odds of exceedance doubles approximately every 10–50 years or 5–20 years under low-end and high-end RCP 8.5 scenarios, respectively (see Fig. 6). The time span required for the odds of exceedance to double (i.e., $\tau$) exhibits less variability across all stations for the 2025–2075 period, relative to the 2000–2050 period, as shown in Fig. 7C, D. This is due to the acceleration of local temperature trends across North America and their tendency to increase faster for higher latitudes (see Extended Data Fig. 1), which leads to more consistency in $\tau$ across all regions. On average, the odds of exceedance doubles every 13 years, while most stations ($\sim$ 96%) are characterized by $\tau <20$ years for the period of 2025–2075 under the intermediate RCP 8.5 scenario.

Under the “business-as-usual” (RCP 8.5) emission scenarios and their projected local warming levels (which, on average until 2100, correspond to $\sim$ 0.39, 0.58, and 0.71 $^{\circ }$C warming per decade for low-end, intermediate, and high-end scenarios, respectively), the odds of exceedance of the 50-year temperature events will still double (possibly multiple times) in the coming decades at some of the stations investigated here, where $\widetilde{\sigma }<1$ $^\circ$C for $\sim$ 90% of the stations. Even though we apply the “business-as-usual” (RCP 8.5) emission scenario for the local temperature projections used here, the exponential nature of the compounding increase in the odds of exceedance of a 50-year temperature event will still likely manifest under less severe emission scenarios (e.g., RCP 4.5), but the doubling time scales will be longer (since the doubling temperature scales remain the same). However, stabilizing global temperatures under low (e.g., RCP 2.6) emission scenarios would suppress the frequency increase in extreme high temperatures to a great extent, according to the stationary analysis presented here.

## Conclusions

Projections of increasing global surface temperature lead to significant transformations in the behavior of high-temperature extremes. Trends in global and local temperatures will transform present-day “once-in-a-lifetime” high-temperature events into frequent (e.g., annual) occurrences in the next several decades. We show that projected temperature trends will exponentially increase the odds of exceeding the 50-year extreme high-temperature events across North America (U.S., Canada, and Mexico) with a doubling time scale of approximately 13–17 years. This doubling time scale is equivalent to a doubling temperature scale of $\sim$ 0.5–1 $^{\circ }$C rise in the local surface temperatures. With the increased frequency of extreme high-temperature events, we expect a corresponding acceleration of many temperature-related public health concerns (see, e.g., ref.^68^). Although it is likely that society has yet to fully comprehend the consequences of global temperature rise, we offer that the information provided here can aid in the development of prevention and mitigation plans at regional and global scales.

## Methods

### Extreme value theory

Extreme Value Theory (e.g., ref.^18^) is a commonly employed method to analyze the occurrence of rare events using probability distributions of a random variable, *x*, which corresponds to the magnitude of an extreme event, i.e., the extreme temperature in the current study. The probability that the event level *x* is exceeded is estimated by *E*(*x*), which is the so-called “exceedance probability distribution” and is given by

1 $$\begin{aligned} E(x)=1-F(x), \end{aligned}$$

where *F*(*x*) is the cumulative probability distribution of the random variable *x*.

Cumulative probability distributions, i.e., $F\left( x\right)$, can be characterized using a variety of statistical models^18^. In the current analysis, we utilize the popular Generalized Extreme Value (GEV) distribution, which is given by

2 $$\begin{aligned} F\left( x;\mu , \sigma , k\right) =e^{-\left( 1+k\left( \frac{x-\mu }{\sigma }\right) \right) ^{-1/k}}, \end{aligned}$$

where $\mu$, $\sigma$, and *k* represent the location (e.g., distribution mean), scale (e.g., standard deviation proxy), and shape (skewness/tail behavior) parameters, respectively^18^. The parameter *k* is of particular importance here since it defines the tail behavior of *F*(*x*). The probability distribution’s tail exhibits an upper bound when $k<0$ and a lower bound when $k>0$. The GEV distribution combines three different families of extreme value distributions based on different *k* parameter values, i.e., Gumbel distribution ($k=0$), Fréchet distribution ($k>0$), and Weibull distribution ($k<0$). Combining Eqs. (1) and (2), the GEV exceedance probability distribution becomes

3 $$\begin{aligned} E\left( x;\mu , \sigma , k\right) =1-F\left( x;\mu , \sigma , k\right) =1-e^{-\left( 1+k\left( \frac{x-\mu }{\sigma }\right) \right) ^{-\frac{1}{k}}}. \end{aligned}$$

**Figure 8:**
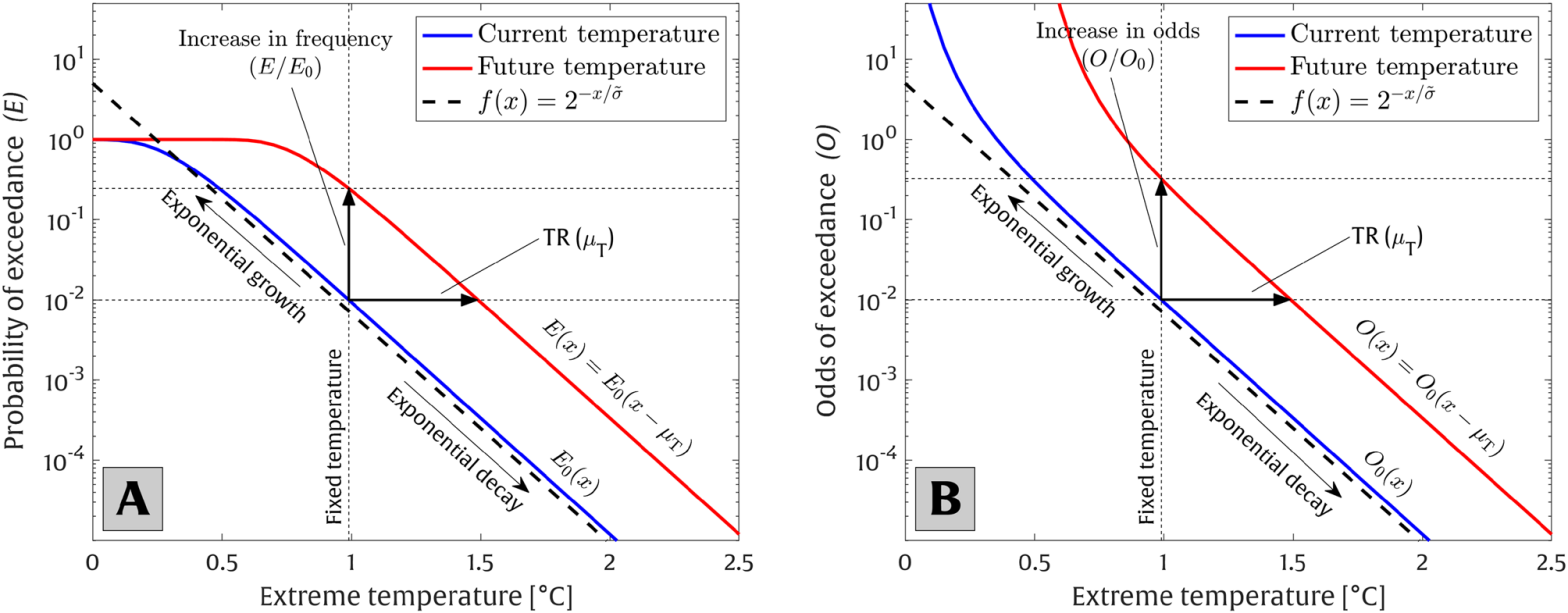
Schematic plots representing the shift in (**A**) the probability of exceedance and (**B**) the odds of exceedance of a particular present-day extreme temperature event under temperature rise ($\mu _{T}$) based on Eqs. (5) and (6), respectively. As evident in panel (**A**), the probability of exceedance is bounded (by 1), while the odds of exceedance, shown in panel (**B**), exhibits an unbounded behavior. In both panels, the values on x- and y-axes are on linear and logarithmic scales, respectively.

Note that since *F*(*x*) is a monotonically increasing function from 0 to 1, *E*(*x*) represents a monotonically decreasing function from 1 to 0. When the shape parameter is close to zero ($k\approx 0$), *E*(*x*) decays exponentially with event level *x* (see Fig. 8A). When the distribution’s mean ($\mu$) increases with time, the probability distribution shifts to the right (see Fig. 8A), causing an increase in the exceedance probability of a specific extreme event. In other words, when the shape parameter is close to zero ($k\approx 0$), the exceedance probability increases exponentially for a fixed event level *x*, subject to an increase in the distribution’s mean (see Fig. 8A).

In the following analysis, which mirrors that of Taherkhani et al.^41^ but for extreme temperature rather than extreme coastal flood level, we adopt a simple exponential form given by

4 $$\begin{aligned} \frac{E}{E_0}=2^\frac{\mu _{T}}{\widetilde{\sigma }}, \end{aligned}$$

to evaluate increases in the exceedance probability (*E*) of an extreme temperature event (with a specific return period, e.g., the 50-year event) subject to a mean temperature increase ($\mu _T$) relative to the unaltered (present-day) distribution ($E_0$).

In Eq. (4), $\mu _{T}$ is the mean temperature rise, and $\widetilde{\sigma }$ is the amount of temperature rise required to double the exceedance probability for a given event level (*x*). For the sake of simplification, we take advantage of the exponential form $2^x$ over $e^x$ [i.e., $\exp (x)$], which clarifies the doubling scale of the exceedance probability. For example, when assuming $\widetilde{\sigma }=2\,^\circ$C, the exceedance probability of a specific extreme temperature event doubles and quadruples under 2 $^{\circ }$C and 4 $^{\circ }$C of temperature rise, respectively. Note that (as discussed in Taherkhani et al.^41^), the variables $\sigma$ and $\widetilde{\sigma }$ are not identical, although they share some similarities in their behavior related to the increasing frequency of extreme events. By assuming that $\widetilde{\sigma }$ remains nearly constant at a location of interest and that $\mu _{T}$ can act as a time-dependent variable derived from local temperature projections, we can obtain estimates of the continuous doubling scale $\widetilde{\sigma }$ by curve fitting the shifted the exceedance probability $(\frac{E}{E_0})$ versus a given temperature rise $(\mu _{T})$ projection for a temperature threshold of interest. Here, to calculate the increase in the frequency of the 50-year extreme temperature event $(x_{50})$, we use shifted (empirical) probability distributions according to the equation

5 $$\begin{aligned} \frac{E}{E_0}=\frac{E_0(x_{50}-\mu _{T})\ }{E_0(x_{50})}, \end{aligned}$$

where $E_0(x_{50}-\mu _{T})$ represents a rightward shift of the present-day distribution $E_0$ by the amount of $\mu _{T}$. Here we apply the 50-year temperature event threshold for our analysis since a 50-year event is nominally equivalent to a ’once-in-a-lifetime’ event. However, other return period events (e.g., 100-year events) often exhibit similarities in terms of their rate of acceleration due to increases in the event mean.

An alternative way to estimate $\widetilde{\sigma }$ is via applying the odds of exceedance of a given event, *O*(*x*), defined as the ratio of the exceedance probability for a given threshold relative to the probability of that event not exceeding that given threshold, instead of the exceedance probability. Mathematically, the odds of exceedance, *O*(*x*), is given by

6 $$\begin{aligned} O\left( x\right) =\frac{E(x)}{1-E(x)}. \end{aligned}$$

Analogous to applying shifted (empirical) exceedance probability distributions, according to Eq. (5), we can write a corresponding shift in the odds of exceedance as

7 $$\begin{aligned} \frac{O}{O_0}=\frac{O_0(x_{50}-\mu _{T})\ }{O_0(x_{50})}, \end{aligned}$$

which is shown in Fig. 8B.

Similar to the increase in the exceedance probability in Eq. (4), the simplified exponential form of doubling behavior in the odds of occurrence can be applied and written as

8 $$\begin{aligned} \frac{O}{O_0}=2^\frac{\mu _{T}}{\widetilde{\sigma }}. \end{aligned}$$

Equation (8) is advantageous over Eq. (4) because the unbounded nature of $O\left( x\right)$ allows for the evaluation of $\frac{O}{O_0}$ under a significantly larger range of $\mu _{T}$, while, on the other hand, *E*(*x*) has the upper bound of 1, which narrows the range over which $\frac{E}{E_0}$ versus $\mu _{T}$ is amenable to curve fitting^41^.

So far, we have considered an exponential form that grows with a shift in the mean ($\mu _{T}$). Alternatively, we can express the exponential growth (in the odds of exceeding an extreme threshold) with time, which is given by

9 $$\begin{aligned} \frac{O}{O_0}=2^\frac{t}{\tau }. \end{aligned}$$

Here, *t* represents the time, and $\tau$ represents a doubling time scale. Like the doubling scale $\widetilde{\sigma }$, the term $\tau$ is location-dependent, and we obtain its value at each station by using shifted empirical distributions of the odds of exceedance subject to local temperature rise projections.

In the current study, we apply temperature projections under a range of probabilistic RCP 8.5 emission scenarios (i.e., the ensemble low-end, intermediate, and high-end RCP 8.5) obtained from the Royal Netherlands Climate Institute (KNMI) Climate Explorer^42^ database, which is based on CMIP5^25^ simulations (https://climexp.knmi.nl/CMIP5/Tglobal/index.cgi) and the World Data Centre for Climate (WDCC)^43^ database, obtained from CMIP6^26^ simulations (https://www.wdc-climate.de/ui/cmip6?input=CMIP6.ScenarioMIP.DKRZ.MPI-ESM1-2-HR.ssp585). The KNMI and WDCC databases provided a total of 15 different global RCP 8.5 projections up to 2300, from which we calculate and apply their ensemble average to utilize as the intermediate RCP 8.5 scenario in our analysis. The low- and high-end RCP 8.5 projections are constructed by taking the ensemble minimum and maximum projections at each time (year) among the 15 projections, respectively. To exclude the inter-annual anomalies present in the temperature projections (which are often associated with climate modes such as ENSO), we apply a low-pass filter (a 4th-order Butterworth low-pass filter with a normalized cut-off frequency of 0.01) to each ensemble time series. Applying the low-pass filter to local temperature projections ensures that the derived growth rates in the odds of exceedance are not dependent on any unknown future phase of ENSO events realized in GCM simulations. Unfiltered temperature projections (with inter-annual variability included) show quite similar (but more noisy) results in terms of the doubling time scale $\tau$ (although this analysis is not shown). This is largely because the doubling time $\tau$ is typically longer (e.g., 5–15 years) than the time scale of many climate modes that control inter-annual variability in regional temperatures.

It is often more intuitive to assess changes in the return period [$T_R(x)$] rather than changes in the exceedance probability or the odds of occurrence at a given threshold. Equation (10) depicts the inverse relationship that exists between $T_R(x)$ and *E*(*x*), i.e., an increase in the exceedance probability results in a decrease in the return period of a given threshold, and it follows as

10 $$\begin{aligned} T_R(x)=\frac{r_i}{E(x)}, \end{aligned}$$

where $r_i$ is the recurrence interval or sampling interval of the observed historical data that are used to obtain the probability distribution. According to the relationship given above, a 1% exceedance probability during a given year (when $r_i=1$ year) is equivalent to an event with a 100-year return period. Thus, under a positive change in the mean temperature ($\mu _{T}>0$), the change in the return period can be calculated as

11 $$\begin{aligned} \frac{T_R \left( x;\mu +\mu _{T},\sigma ,k \right) }{T_R(x;\mu ,\sigma ,k)}= \left( \frac{E\left( x;\mu +\mu _{T},\sigma ,k\right) }{E\left( x;\mu ,\sigma ,k\right) }\right) ^{-1}, \end{aligned}$$

which means that any potential form of exponential growth in the exceedance probability (or, similarly, odds) at a given threshold corresponds to an exponential decay in the return period.

### Data and application

NOAA’s National Centers for Environmental Information (NCEI) archives the hourly/daily temperature observations across North America (U.S., Canada, and Mexico) in the NOAA-GHCND database (https://www.ncei.noaa.gov/products/land-based-station/global-historical-climatology-network-daily). For the current analysis, we obtained these temperature data set via their API data access protocols. The hourly/daily maximum temperature record for 18,698 stations across U.S., Canada, and Mexico were downloaded, with the longest records containing data from 1840 to 2018 (178 years). All stations that provided only hourly temperature records were converted to daily maximum temperature records for the analysis described below. Only stations with more than 50 years of data and at least 80% data coverage are retained in this analysis. Additionally, stations with less than 80% coverage during summer periods (June-July-August) were removed from the analysis. This process filters out $\sim$77% of the stations initially considered, leaving 4266 stations with high-quality data that we used in the present analysis. On average, the length of available historical data for these stations is 88 years. Similar to Taherkhani et al.^41^, to obtain the parameter estimates for the GEV distribution (2), we apply the top three annual maxima (i.e., $r_i=1/3$; block size of 1 year for extreme event selection) from the set of available temperature observations. Annual block sizes are found to be reasonable for the analyses of high-temperature extremes^10,11^. To eliminate the effect of local warming trends on the magnitude and frequency of extreme high temperatures, we detrended (linearly) the time series of historical temperatures. The choice of the top three annual events ensures that the influence of large, isolated extreme high-temperature events is restricted.

GEV has proven to be a robust model for analyzing temperature extremes; however, it is not without limitations (see, e.g., ref.^23^). GEV is highly sensitive to the length of the available observational data, where limited records return less reliable estimates of extreme events. However, the GEV model is generally capable of assessing the statistical behavior of rare events (e.g., a 50-year event) even when the observational time series available is shorter than the return period of those events (i.e., 50 years)^10,23^. Fortunately, in the case of our analysis, all stations have records of 50 years of data or more, lending confidence to the estimates of GEV model parameters.

We examine two different scenarios to assess transformations of extreme temperature events. In the first scenario, we investigate the potential temperature increase that would cause the present-day 50-year event to become exceeded annually, which is calculated as follows

12 $$\begin{aligned} \Delta T_{50yr\rightarrow 1yr}=x\left( T_R=50;\;\mu ,\sigma ,k\right) -x\left( T_R=1;\mu ,\sigma ,k\right) , \end{aligned}$$

where $\Delta T_{50yr\rightarrow 1yr}$ is the difference in temperature extremes corresponding to a 50-year, $x\left( T_R=50\ ;\mu ,\sigma ,k\right)$, and a 1-year (annual), $x\left( T_R=1;\mu ,\sigma ,k\right)$, temperature event. Both 50-year $(T_R=50)$ and 1-year $(T_R=1)$ temperature events are obtained by inverting the GEV distribution at each location. As shown below in Fig. 2A, the value of $\Delta T_{50yr\rightarrow 1yr}$ is typically $\sim$2-7 $^\circ$C across North America (see section “Results” for more details).

In the second scenario, we inspect the temperature rise needed to cause a shift from an annual event to the nominal mean summer temperature (MST) level, according to the equation

13 $$\begin{aligned} \Delta T_{1yr\rightarrow MST}=x\left( T_R=1;\;\mu ,\sigma ,k\right) -MST. \end{aligned}$$

Here, similar to Eq. (12), $\Delta T_{1yr\rightarrow MST}$ represents the difference between the annual temperature event, $x\left( T_R=1;\;\mu ,\sigma ,k\right)$, and MST, which is defined as the average of the daily maximum temperature in summer (June-July-August) from the (linearly detrended) time series of the observed hourly/daily temperatures at each station. As shown below in Fig. 2B, the value of $\Delta T_{1yr\rightarrow MST}$ is typically $\sim$2–12 $^\circ$C across North America.

Having calculated the required temperature difference for each station under the two above-mentioned scenarios, we find the time (year) in the future that each of these two scenarios is expected to take place for each location based on projections of local temperature rise under a range of RCP 8.5 emissions scenarios (described in section “Extreme value theory”).

### Supplementary Information

Supplementary Figures.

Supplementary Information.

## Data Availability

The data and programming codes required to reproduce this analysis are publicly available at https://github.com/adavaria/temperature-extremes.
