# Supplementary material for: Exponential increases in high-temperature extremes in North America
Source: Sci Rep. 2023 Nov 6;13:19177. doi: 10.1038/s41598-023-41347-3 (PMC10628173; doi:10.1038/s41598-023-41347-3)
Supplement: Supplementary file 1 — Supplementary Figures. [file 41598_2023_41347_MOESM1_ESM.pdf]

# Extended Data

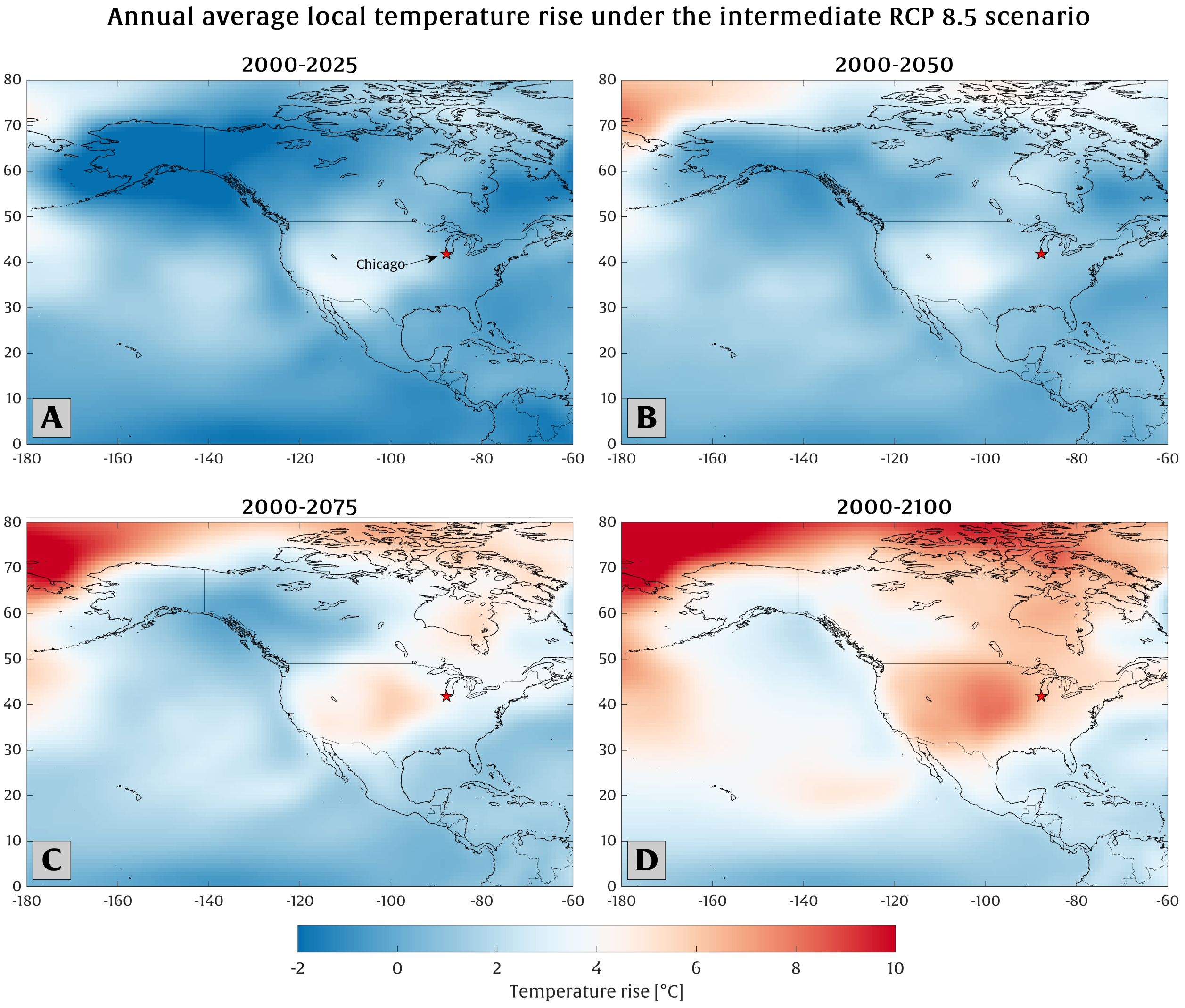


**Figure 1:** Annual average warming (local temperature rise) projections (WDCC [46]) under the intermediate RCP 8.5 emission scenario across North America during (A) 2000-2025, (B) 2000-2050, (C) 2000-2075, and (D) 2000-2100. The baseline for the local temperatures is the annual average local temperature during the year 2000. The intermediate RCP 8.5 scenario projects a global average temperature rise of ∼0.1, 1.2, 2.4, and 4°C by 2025, 2050, 2075, and 2100 relative to 2000, respectively, alluding that the local warming trends outpace the global average throughout the majority of North America.

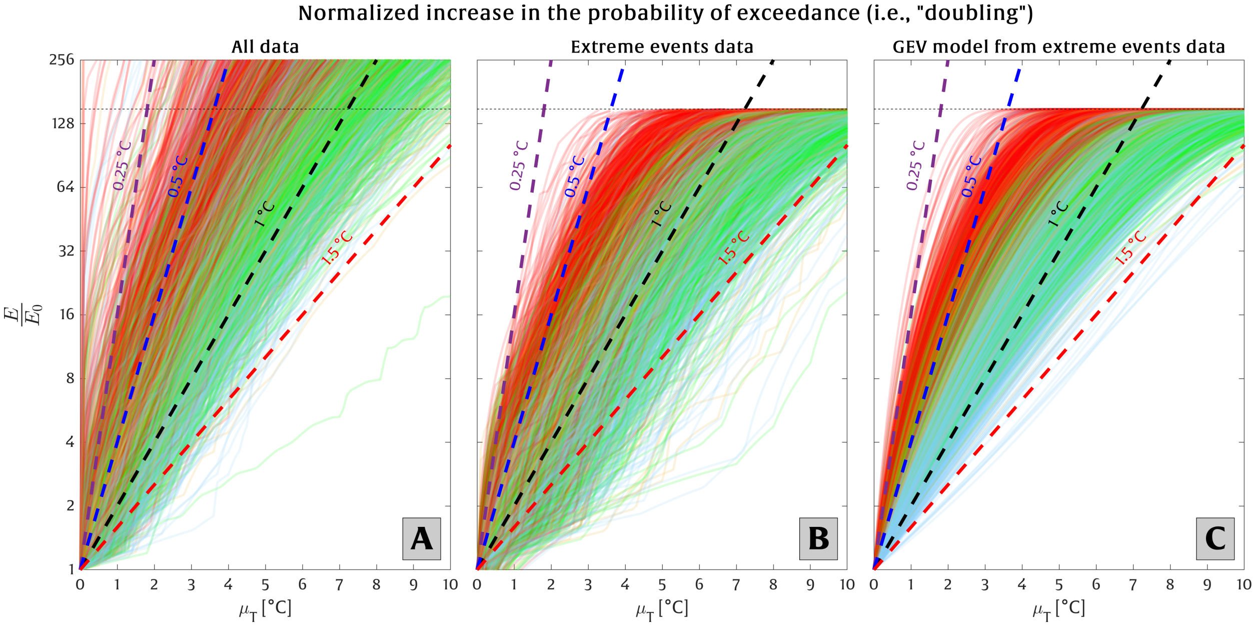


**Figure 2:** The relative increase in the probability of exceedance of the present-day 50-year extreme temperature event ($E/E_0$) under temperature rise ($\mu_T$). Here, $E_0$ denotes the present-day exceedance probability of a 50-year temperature extreme, and $E = E_0(x - \mu_T)$ represents the future (shifted) exceeding probability of that event. $E_0$ is calculated via three approaches. Panel A depicts the relative increase in the exceedance probability by using all the recorded hourly/daily temperatures ($x$ represents all recorded data). However, panels B and C evaluate $E/E_0$ by only incorporating the top 3 annual maxima ($r_i = 1/3$) of the recorded temperatures ($x$ represents the top 3 annual extreme events). Panel B uses the empirical (i.e., using recorded data) exceedance probabilities, and panel C uses the best-fit GEV-modeled exceedance probabilities. The x- and y-axes are on linear and logarithmic (base two) scales, respectively, meaning that the straight lines in each panel demonstrate an exponential growth rate in the exceedance probability. Each solid line in these panels indicates a climate station, which is color-coded according to its corresponding cluster (based on its GEV model parameters) shown in Fig. 1. The purple, blue, black, and red dashed lines depict the doubling of the probability of exceedance under every 0.25, 0.5, 1, and 1.5°C rise in local temperatures. Note that the jaggedness of the solid lines in panels A and B arises from the use of recorded observations, while the GEV model-derived probabilities of exceedance provide smooth lines in panel C. Also, since the probability of exceedance is limited to 1 eventually ($E \to 1$), the relative increase in the probability of exceedance is bounded according to Eq. (10) (i.e., $E/E_0 = 1/E_0 = 1/(r_i/T_R) = T_R/r_i = 50/(1/3) = 150$) when using temperature extremes in panels B and C. By comparing these panels, it is evident that the growth rates in the exceedance probabilities are to a large extent independent of the form of the exceedance distribution applied here.

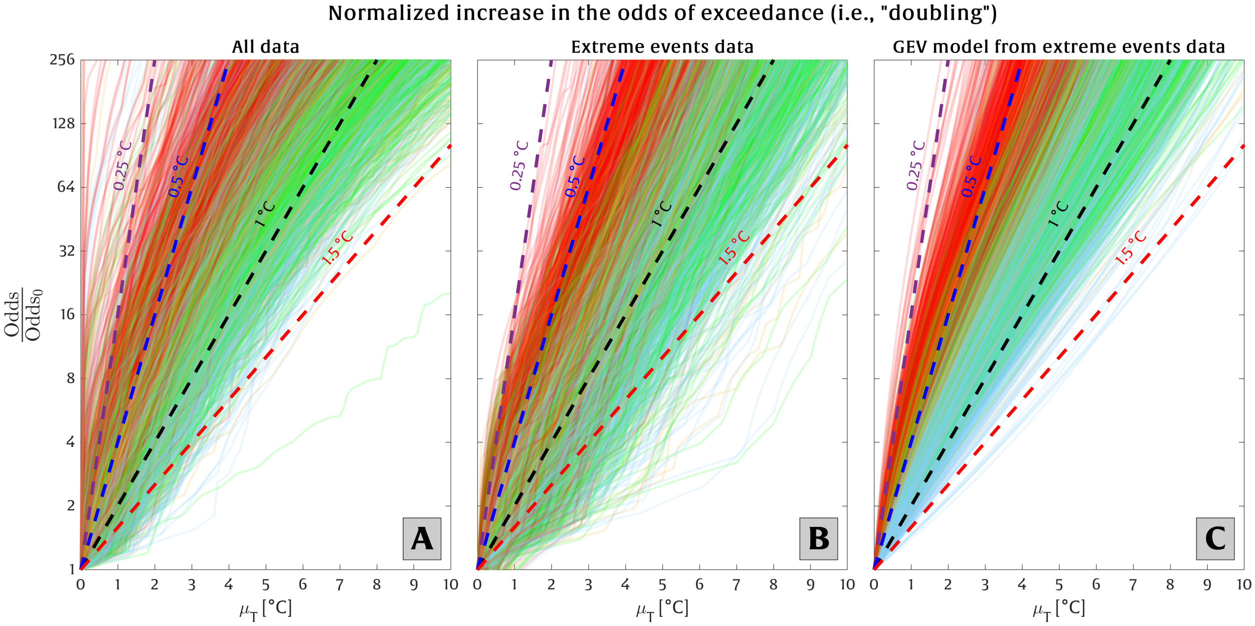


**Figure 3:** The relative increase in the odds of exceedance of a present-day 50-year extreme temperature event ($O/O_0$) under temperature rise ($\mu_T$) according to the relationship between the odds and probability of exceedance [Eq. (6); $O = E/(1-E)$]. Similar to Fig. 2 in Extended Data, $E_0$ denotes the present-day exceedance probability of a 50-year temperature extreme, and $E = E_0(x - \mu_T)$ represents the future (shifted) exceeding probability. Here, similarly, $O_0$ is calculated via three approaches. Panel A depicts the relative increase in the odds of exceedance by using all the recorded hourly/daily temperatures ($x$ represents all recorded data). However, panels B and C evaluate $O/O_0$ by using only the top 3 annual maxima ($r_i = 1/3$) of the recorded temperatures ($x$ represents the top 3 annual extreme events). Panel B applies the empirical (i.e., using recorded data) distribution of the odds of exceedance, and panel C uses the best-fit GEV-modeled odds of exceedance distribution. The x- and y-axes are on linear and logarithmic (base two) scales, respectively, meaning that the straight lines on each panel demonstrate an exponential growth rate in the odds of exceedance. Each solid line in these panels represents a climate station, which is color-coded according to its corresponding cluster (based on its GEV model parameters) shown in Fig. 1. The purple, blue, black, and red dashed lines depict the doubling of the odds of exceedance under every 0.25, 0.5, 1, and 1.5°C rise in local temperatures. Note that the jaggedness of the solid lines in panels A and B arises from the use of observed data, while the GEV model-derived odds of exceedance are smooth. In contrast to panels B and C in Fig. 2 in Extended Data, since the odds of exceedance is unbounded [based on Eq. (6)], the relative increase in the odds of exceedance is unbounded as well. By comparing these panels, it is evident that the growth rates in the exceedance odds are, to a large extent, independent of the form of the exceedance distribution adopted. In general, Figs. 2 and 3 in Extended Data represent almost identical trends in the growth of probabilities and odds of exceedance versus temperature rise.

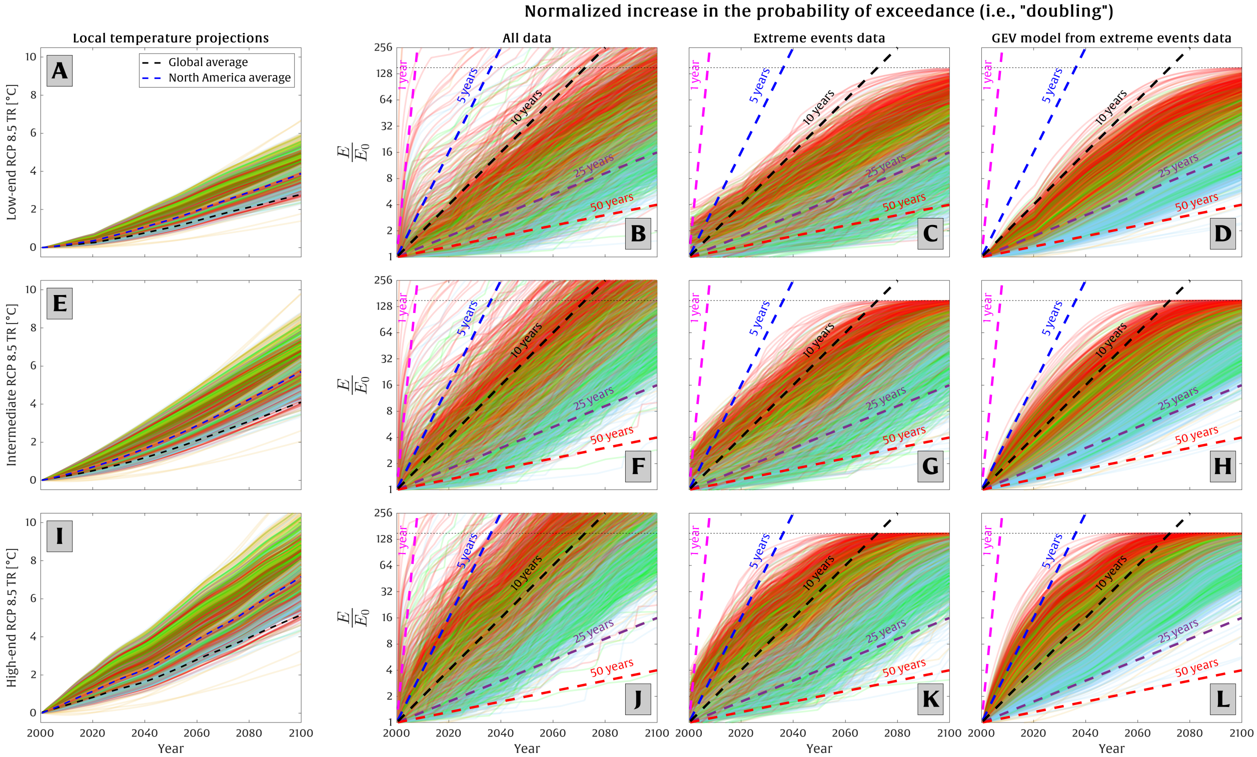


**Figure 4:** The relative increase in the probability of exceedance of the present-day 50-year extreme temperature event ($E/E_0$) with respect to time, where local temperature rise projections under RCP 8.5 scenarios provided by KNMI [64] and WDCC [46] are utilized and shown in panels A, E, and I. Similar to Fig. 2 in Extended Data, $E_0$ denotes the present-day exceeding probability of a 50-year temperature extreme, and $E = E_0(x - \mu_T)$ represents the future (shifted) exceeding probability of that event. $E_0$ is calculated via three approaches under each RCP 8.5 scenario. Panels B, F, and J depict the relative increase in the exceedance probability by using all the recorded hourly/daily temperatures ($x$ represents all recorded data). However, panels C, D, G, H, K, and L evaluate $E/E_0$ by only using the top 3 annual maxima ($r_i = 1/3$) of the recorded temperatures ($x$ represents the top 3 annual extreme events), whereas in panels C, G, and K use empirical exceedance probabilities (i.e., using recorded data) and in panels D, H, and L use best-fit GEV-modeled exceedance probabilities. The x- and y-axes are on linear and logarithmic (base two) scales, respectively, meaning that the straight lines in each panel demonstrate an exponential growth rate in the exceedance probability. Each solid line in these panels stands for a climate station and is color-coded according to its corresponding cluster (based on its GEV model parameters) shown in Fig. 1. The pink, blue, black, purple, and red dashed lines depict doubling the probability of exceedance every 1, 5, 10, 25, and 50 years, respectively, into the future. Note that the jaggedness of the solid lines in panels B, C, F, G, J, and K arises from using observed data, while the GEV model-derived probabilities are smooth in panels D, H, and L. Also, since the probability of exceedance is limited to 1 eventually ($E \to 1$), the relative increase in the probability of exceedance is bounded according to Eq. (10) (i.e., $E/E_0 = 1/E_0 = 1/(r_i/T_R) = T_R/r_i = 50/(1/3) = 150$) when using temperature extremes in panels C, D, G, H, K, and L. By comparing the panels in each row (under each RCP 8.5 scenario), it is evident that the growth rates in the exceedance probability are largely independent of the form of the exceedance distribution adopted.

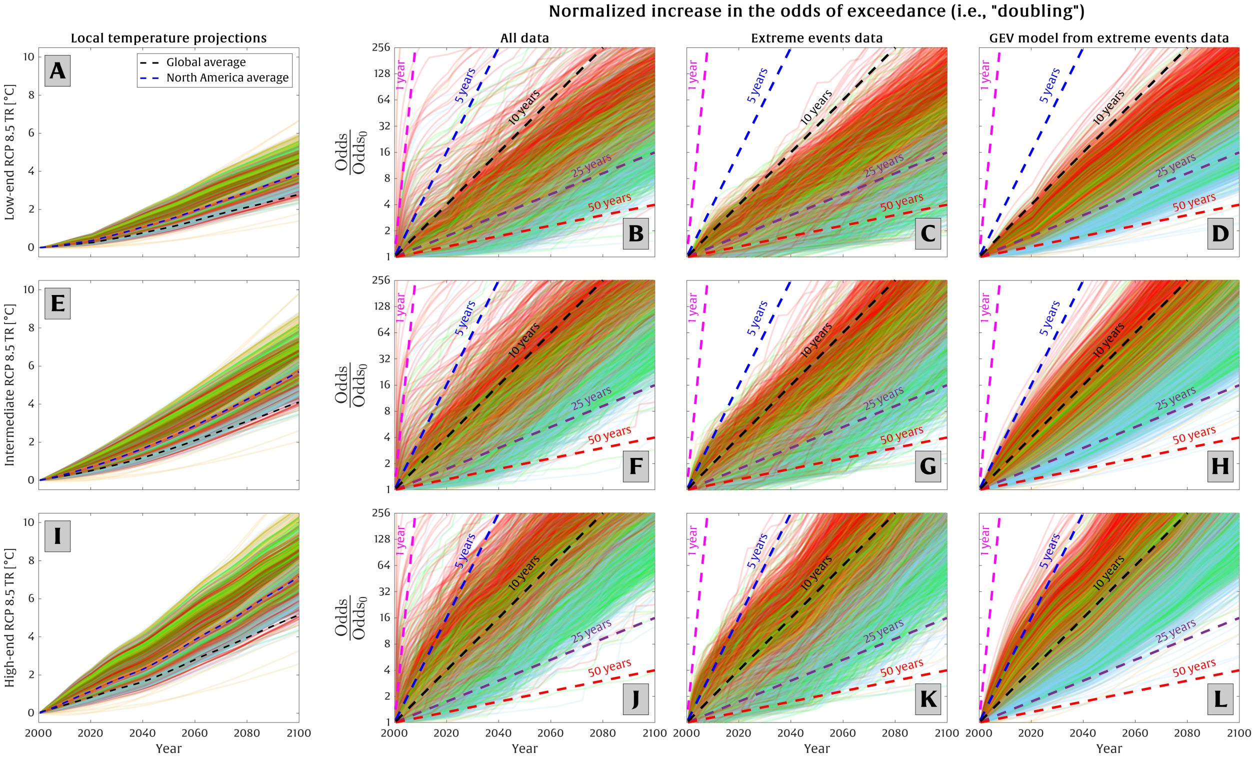


**Figure 5:** The relative increase in the odds of exceedance of the present-day 50-year extreme temperature event $(O/O_0)$ according to the relationship between the odds and probability of exceedance [Eq. (6); $O = E/(1-E)$] with respect to time, where local temperature rise projections under RCP 8.5 scenarios provided by KNMI [64] and WDCC [46] are utilized and shown in panels A, E, and I. Similar to Fig .4 in Extended Data, $O_0$ denotes the present-day odds of exceedance distribution of a 50-year temperature extreme, and $O = O_0(x - \mu_T)$ represents the future (shifted) odds of exceedance. $O_0$ is calculated via three approaches under each RCP 8.5 scenario. Panels B, F, and J depict the relative increase in the odds of exceedance by using all the recorded hourly/daily temperatures ($x$ represents all recorded data). However, panels C, D, G, H, K, and L evaluate $O/O_0$ by only using the top 3 annual maxima ($r_i = 1/3$) of the recorded temperatures ($x$ represents the top 3 annual extreme events). Panels C, G, and K use the empirical (i.e., using recorded data) odds of exceedance, and panels D, H, and L use the best-fit GEV-modeled odds of exceedance. The x- and y-axes are on linear and logarithmic (base two) scales, respectively, meaning that the straight lines on each panel demonstrate an exponential growth rate in the odds of exceedance. Each solid line in these panels denotes a climate station, which is color-coded according to its corresponding cluster (based on its GEV model parameters) shown in Fig. 1. The pink, blue, black, purple, and red dashed lines depict the doubling of the odds of exceedance every 1, 5, 10, 25, and 50 years, respectively, into the future. Note that the jaggedness of the solid lines in panels B, C, F, G, J, and K arises from using observed data, while the GEV model-derived odds of exceedance curves are smooth in panels D, H, and L. In contrast to Fig. 4 in Extended Data, since the odds of exceedance is unbounded [based on Eq. (6)], the relative increase in the odds of exceedance is unbounded as well. By comparing the panels in each row (under each RCP 8.5 scenario), it is evident that the growth rates in the exceedance odds are largely independent of the form of the exceedance distribution adopted. In general, Figs. 4 and 5 in Extended Data represent almost identical trends in the growth of probabilities and odds of exceedance versus time.

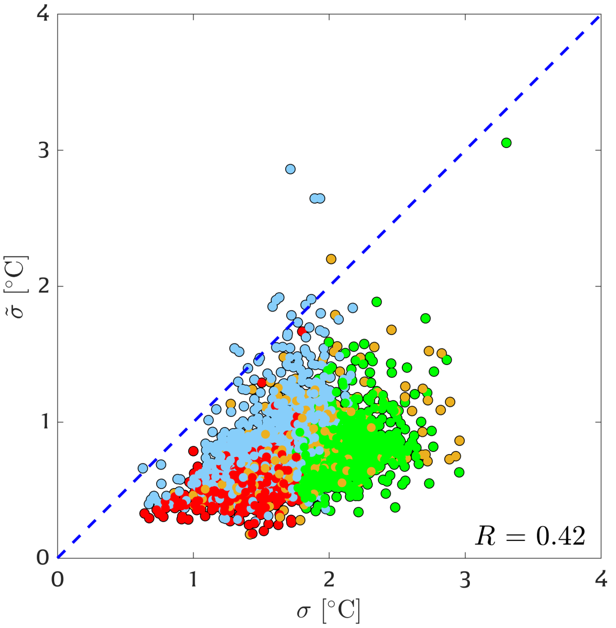


**Figure 6:** The relationship between the amount of local temperature rise required to double the odds of exceedance of the present-day 50-year extreme temperature event ($\widetilde{\sigma}$; see Eq. (8)) and the GEV model's scale parameter ($\sigma$). Each dot is associated with a climate station, which is colored according to its corresponding cluster (based on its GEV model parameters) shown in Fig. 1. Overall, the correlation between these two metrics ($\widetilde{\sigma}$ and $\sigma$) is modest ($R = 0.42$), where the blue dashed line shows a one-to-one ($R = 1$) relationship between them. However, the stations showing almost identical $\widetilde{\sigma}$ and $\sigma$ values are represented by the color blue (i.e., the stations with $k \approx 0$).
